# Using Variational Autoencoders for Out of Distribution Detection in Histological Multiple Instance Learning

**DOI:** 10.1109/access.2025.3593420

**Published:** 2025-07-28

**Authors:** FRANCISCO JAVIER SÁEZ-MALDONADO, LUZ GARCÍA, LEE A. D. COOPER, JEFFERY A. GOLDSTEIN, RAFAEL MOLINA, AGGELOS K. KATSAGGELOS

**Affiliations:** 1 Department of Computer Science and Artificial Intelligence, Universidad de Granada, 18071 Granada, Spain; 2 Department of Signal Theory, Telematics, and Communications, 18071 Granada, Spain; 3 Center for Computational Imaging and Signal Analytics, Northwestern University, Chicago, IL 60611, USA; 4 Chan Zuckerberg Biohub Chicago, Chicago, IL 60642, USA; 5 Department of Pathology, Northwestern University, Chicago, IL 60611, USA; 6 Department of Electrical and Computer Engineering, Northwestern University, Chicago, IL 60611, USA

**Keywords:** Out-of-distribution detection, multiple instance learning, variational autoencoder

## Abstract

In the context of histological image classification, Multiple Instance Learning (mil) methods only require labels at Whole Slide Image (wsi) level, effectively reducing the annotation bottleneck. However, for their deployment in real scenarios, they must be able to detect the presence of previously unseen tissues or artifacts, the so-called Out-of-Distribution (ood) samples. This would allow Computer Assisted Diagnosis systems to flag samples for additional quality or content control. In this work, we propose an ood-aware probabilistic deep mil model that combines the latent representation from a variational autoencoder and an attention mechanism. At test time, the latent representations of the instances are used in the classification and ood detection tasks. We also propose a deterministic version of the model that uses the reconstruction error as ood score. Panda (prostate tissue) and Camelyon16 (lymph node tissue) are used as train/test in-distribution datasets, obtaining bag classification results competitive with current state-of-the-art models. ood detection is evaluated performing two experiments for each in-distribution dataset. For Panda, Camelyon16 and artif (prostate tissue contaminated with artifacts) are used as ood datasets, obtaining 100% auc in both cases. For Camelyon16, Panda and bcell (lymph node tissue diagnosed with diffuse large B-cell lymphoma) are used as ood datasets, obtaining aucs of 100% and 97%, respectively. Experimental validation demonstrates the models’ strong classification performance and effective ood slide detection, highlighting their clinical potential.

## I INTRODUCTION

Multiple Instance Learning (mil) is a weakly-supervised learning approach that has recently gained enormous popularity [1], [2]. mil drastically reduces the annotation effort [3], which is the main bottleneck in many medical Computer Aided Diagnosis (cad) systems. In mil, each element in the training set is called a bag, and it is composed of multiple instances. Under the standard mil assumption [4], each instance has a hidden binary class label, and a bag is positive if, and only if, one or more of its instances are positive. Although different mil assumptions have been proposed in the literatures [5] and [6], the standard assumption of a hidden binary label per instance is the most frequently used [4].

mil methods are faced with the task of correctly classifying the bag and possibly the instances within the bag while only using bag labels. This is the case of histological image classification, where a frequently sought goal is to determine whether a Whole Slide Image (wsi) contains tumorous tissue [7]. In this case, the wsi is considered the bag, and the instances are small patches from the slide.

There exist two main approaches to designing a mil classifier: instance-based mil, where the individual instances are considered to contain the discriminative information for the classification [8]; and embedding-based mil, where the information extracted from the instances is combined to create a richer representation of the whole bag to be classified. See [9] for a recent and clear presentation of mil approaches. In practice, embedding-based models have shown superior performance in the classification task. The main reason for this is that aggregating the information from all the instances produces a regularized bag representation which facilitates the classification task [9], [10]. Therefore this approach is the most frequently used in the recent literature.

Most of the state-of-the-art (sota) embedding-based deep mil models utilize an attention mechanism. The first was proposed in [11] and is known as Attention-Based mil (abmil). This model creates permutation-invariant bag representations using the importance of each instance for the classification task. Usually, this results in positive instances in the bag having higher attention values than negative ones, providing an interpretable output of the model. mil models based on an attention mechanism have evolved a lot since abmil was presented, refining their predictive metrics in a variety of ways, such as introducing instance correlations [9], [12], [13], using two branches to further detect key instances [14], [15], or introducing mathematical operators that smooth the attention values along neighbour instances [16], [17].

Although the accuracy of current sota deep mil methods in the classification task is very high, they fail at test time when the input to the model does not have the same structural or morphological features as the training data [18], [19]. In this work, we follow [20], which describes anomaly or Out-of-Distribution (ood) detection as the process of identifying the samples that do not belong to the training distribution (IN-Distribution, ind).

In digital pathology, detecting ood samples, either at bag or instance level, is of crucial importance, since flagging a sample as ood alerts pathologists about the ignorance of the model on the input data. In a real-world scenario, it is common to find slides that contain secondary tumours unseen during training. Tissue cross-contamination also occurs, some instances in the bag come from a different tissue. Furthermore, other artifacts such as blood, folds, or blur can appear [21], [22]. The ood literature distinguishes between Near and Far ood problems, which are characterized by their difficulty. Following [23], in Near-OOD, the outlier and inlier classes are highly similar, while in Far-OOD, the outlier is more distinct from the training distribution [24], [25].

The frequent appearance of ood samples at test time poses an important challenge to mil models since, to the best of our knowledge, they *know what they know* but, unfortunately, *they don’t know what they don’t know*. Since they are trained under the closed-world assumption with ind samples, they expect test data to be drawn independently from the same distribution. The main reason for the lack of ood awareness of current deep mil methods is that they do not model the underlying data distribution in the training set. Because of this, current mil models can only use model-agnostic ood scores like entropy [23] or max-logit [26], which are not trained in the specific data distribution. While the existing literature on the use of mil in histological image classification continues to grow [1], surprisingly, little attention has been paid to the use of techniques that provide current mil methods with the ability to model the data distribution.

In this work, we tackle the mil classification and ood detection problems by using a deep generative model coupled with a mil method. To be precise, we use a Variational Autoencoder (vae) which explicitly models the data distribution and calculates the likelihood of any given instance. The probabilistic latent representations of the instances obtained from the vae are used in an Attention-Based mil (abmil, [11]) to classify ind bags. Furthermore, those representations are used to compute the marginal likelihood of the instances, which provides the basis for the calculation of a probabilistic ood score. We name our method vaeabmil. We also present a deterministic version of vaeabmil, named daeabmil, in which the probabilistic representation is replaced by its deterministic version that is simpler to optimize.

We apply the proposed models to two classification tasks using two well-known datasets: Camelyon16 and Panda. We then present two Far-ood detection setups, in which the ind and ood slides do not share the main organ type. Finally, we present two Near-ood detection experiments using the bcell and artif datasets (only used as ood data), in which the ind and ood slides share the main type of tissue (breast and prostate tissue, respectively). We show that the classification performance of vaeabmil and daeabmil is similar to that of the sota deep mil models for ind data. Furthermore, the ood detection experiments show that our models excel at detecting ood samples. This constitutes the main benefit of using vaeabmil and daeabmil: while achieving competitive results in bag-level classification, they are in addition able to determine which bags do not belong to the original ind dataset, which is a task that the rest of the sota models are not designed to perform. Our proposals are in fully agreement with [23]: ood detection is a capability Computer Assisted Diagnosis (cad) systems should be provided with. We achieve it by making use of the latent representation produced by our models.

In summary, our contributions are the following:

- We introduce vaeabmil, a novel probabilistic deep mil method that combines a vae with abmil to perform ind classification and bag-level ood detection. We also propose a deterministic version of the model, named daeabmil, which shows optimization benefits. vaeabmil and daeabmil constitute the first mil models with trainable ood scores.
- We perform an extensive experimentation to validate and show the benefits of our proposal. We use Panda and Camelyon16 as train-test in-distribution datasets. vaeabmil and daeabmil obtain competitive bag classification results with current sota mil models.
- In the ood detection task, vaeabmil and daeabmil and their respectively tailored ood scores logpx and recerr, are exhaustively compared against sota mil models using model-agnostic ood scores. Notice that, so far, no tailored ood scores have been defined for them. A statistical significance analysis on ood performance is also included.
- Additionally, we provide a deep analysis of the impact of two different feature extractors in the classification and ood detection metrics. We experimentally show, for the first time in the ood-mil literature, the benefits of using a foundation model for detecting ood bags in mil problems.

The rest of the paper is organized as follows. In Section II we first describe the related ood detection work in digital pathology and then we provide an overview of the abmil method and variational autoencoders. In Section III we present vaeabmil first, then daeabmil (Section III-A), and lastly we introduce the proposed ood scores for both methods (Section III-C). The experiments are presented in Section IV, followed by the conclusions drawn from this work which are explained in Section V. Lastly, further experimental analysis is provided in Appendices A and B.

## II BACKGROUND

In this section, we present the related work (Section II-A). We then mathematically formulate the mil problem and describe the tools that provide the basis for constructing our mil method with ood capabilities (Section II-B).

### A. RELATED WORK

The popularity of mil in digital pathology has grown exponentially due its benefits in wsi classification. See [1], [2], [27] for recent reviews of the sota methods.

Out-of-distribution detection undoubtedly plays a very important role in computational pathology [18], [28], [29], reflected by an increasing number of contributions. For instance, [30] provides a comparative analysis of few-shots-exposure and unsupervised uncertainty estimation techniques, proposing a cosine distance-based ood detection approach for retinal OCT images. Notice that other reconstruction errors can also be used [31]. Deterministic uncertainty estimations of classifiers and ensembles for ood threshold-based detection are presented in [32] in the framework of breast and prostate cancer detection in histopathological images. Furthermore, a probabilistic uncertainty estimation is proposed in [33] using a Bayesian U-net to detect anomalies in OCT images. In [21], a deep kernel model is used to detect histological artifacts, blur, and folds in glass slides of bladder tumour resections. Lastly, the most recent works review the use of AnoDDPM [34] and AnoLDM [35] for ood detection in digital pathology. However, none of the previously mentioned works are developed under the mil framework.

The importance of using a good latent representation of the data has been widely acknowledged in different research areas, see, for instance [36] and [37]. vaes provide a good example of it and they have been frequently used for standalone ood detection [38], [39], [40]. In the medical domain, they have been used for unsupervised anomaly localization in CT scans [41] or anomaly detection in electrocardiogram records [42], always outside the mil paradigm. In [43], a VAE is used to define a mil model that creates a disentangled representation of the instance features, later used for ood generalization: the task in which the model is used on samples from another dataset and is expected to maintain its classification performance. Note that ood generalization is not the same task as ood detection, so [43] does not propose a mil based ood model. Thus, the use of vaes for ood detection in the mil framework remains, so far, unexplored.

The aggregation of instance-level ood scores to perform ood detection is explored in [23], where multiple patch-level CNNs are trained and the patch-level entropy is aggregated to obtain a bag-level ood score. Notice that this is not a mil classification model but the use of the estimated patch-level classification probabilities to define a bag-level ood score.

To conclude this section, we remark that although recent references on the use of ood detection methods in wsi classification exist, none of them has been formulated using the mil paradigm. Our vaeabmil and daeabmil constitute pioneering approaches on providing mil methods with ood capabilities.

### B. DEEP MULTIPLE INSTANCE LEARNING

Our work focuses on embedding ood detection capabilities in deep MIL classification models. For this reason, we start by presenting the elements of the mil setup for the classification task. In MIL, each element of the dataset is a pair $\left(X,\;y\right)$, where $X\;\in \;{\mathbb{R}}^{N_{b}\times P}$ is a bag with P∈ℕ the dimension of the feature space provided by a pretrained encoder, and y is the bag label. Each bag is composed of $N_{b}$ instances, $X={\left[x_{1},\;\cdots ,\;x_{N_{b}}\right]}^{T}$. In this work, we consider a binary classification problem. Following the so-called standard mil assumption [4], a bag is positive if, and only if, at least one of its instances is positive. That is, $y=\max{\left\{y_{i}\right\}}_{i=1}^{N_{b}}\;\in \;\left\{0,\;1\right\}$, where $y_{i}$ is the label of the instance $x_{i}$. We consider our dataset to have B pairs, and we will use the notation $X^{b}$ to denote the b−th bag of the dataset, with instances $x_{1}^{b},\;\cdots ,x_{N_{b}}^{b}$. Unless necessary, we will omit the bag reference $\left(b\right)$ for simplicity.

The goal in the classification task is to learn a function that maps each input bag to a label. At test time, a previously unseen bag $X^{★}$ is received by the model which outputs a class for it. As indicated in the introduction, we will follow the *embedding-based* approach to designing a mil classifier that solves the standard mil problem. The model creates a representation B of the bag by aggregating the information of its instances and uses it to assign a label to each bag. To create the aforementioned representation, the current most relevant models are *deep attention* mil models. These methods are composed of three main blocks: a feature refiner, an attention mechanism and a classifier. We now describe each of the blocks individually.

First, in deep attention mil models, each instance $x_{i}$ of the bag is processed using a neural network $g_{\eta }$, the feature refiner, with parameters η. This creates a latent representation of that instance $z_{i}=g_{\eta }\left(x_{i}\right)\;\in \;{\mathbb{R}}^{D}$, with D∈ℕ the latent space dimension, which contains its most relevant information. We denote by $Z^{T}=\left[z_{1},\;\cdots ,\;z_{N_{b}}\right]$ the matrix containing the latent representations of the instances in a bag.

In embedding-based MIL, the information of the instances is aggregated to create a richer representation of the whole bag that takes into account *how important each instance is in the bag representation*. This importance value is often called *attention value*, and it is widely used in many current deep mil models such as abmil [11], transmil [12] or dtfdmil [9]. In this work, we build upon the well known abmil model, in which the attention module computes the vector of attention values f as follows: considering $W\;\in \;{\mathbb{R}}^{L\times D}$ and $w\;\in \;{\mathbb{R}}^{L}$ to be learnable weights and L∈ℕ,

(1) $$F_{\text{mid}}=\tanh\left(ZW^{T}\right)\;\in \;{\mathbb{R}}^{N_{b}\times L}$$

(2) $$f=F_{\text{mid}}w\;\in \;{\mathbb{R}}^{N_{b}}.$$

The softmax is applied to f to obtain the attention values that are all positive and add up to one. Then, each obtained value is multiplied by its corresponding embedding and aggregated to obtain the final bag representation B as:

(3) $$B\;:=Z^{T}\;\text{Softmax}\left(f\right)\;\in \;{\mathbb{R}}^{D}$$

This bag representation aggregates the information of the instances of the bag according to their importance in the classification task. Finally, we pass it through a simple linear classifier $c_{\gamma }\;:\;{\mathbb{R}}^{D}\;\to \;\left[0,\;1\right]$ with parameters γ, which assigns to each bag its probability of being of the positive class.

### C. VARIATIONAL AUTOENCODERS

The usage of vaes in mil is the key proposal of our work. In VAEs, instead of considering a single, deterministic latent representation z for each input, they place a prior distribution $p\left(z\right)$ over that latent encoded representation. Given z, a probabilistic reconstruction is obtained using an observation model $p\left(x|z\right)$. Typically, the prior is chosen to be a standard Gaussian distribution $p\left(z\right)=N\left(\text{0},\;I\right)$ since it enforces smoothness, as well as beneficial structural and continuity properties in the latent space. The observation model is also chosen Gaussian $p_{\theta }\left(x|z\right)=N\left(x|m_{\theta }\left(z\right),\sigma_{\theta }^{2}\left(z\right)I\right)$, where the mean function $m_{\theta }\left(z\right)$ and covariance $\sigma_{\theta }^{2}\left(z\right)I$ are parameterized by neural networks with parameters θ.

With this selection of the prior and likelihood distributions, predictions in vaes are made by integrating over the posterior distribution $p\left(z|x\right)$ which, unfortunately, can not be computed in closed form. For this reason, Variational Inference (VI) [44] is often used as a form of approximating the exact posterior using a Gaussian variational distribution $q_{\phi }\left(z|x\right)$

(4) $$p\left(z|x\right)\;\approx \;q_{\phi }\left(z|x\right)=N\left(z|m_{\phi }\left(x\right),\sigma_{\phi }^{2}\left(x\right)I\right),$$

where the mean and the covariance are parameterized by neural networks ($m_{\phi }\left(x\right)$ and $\sigma_{\phi }^{2}\left(x\right)$, respectively) with parameters ϕ. To optimize the parameters of the likelihood and posterior distributions we maximize the Evidence Lower Bound (elbo) [45], which lower bounds the marginal likelihood of the data $p\left(x\right)$. The elbo in vaes for a sample x takes the form:

(5) $$L_{\phi ,\theta }^{\text{VAE}}\left(x\right)=E_{q_{\phi }\left(z|x\right)}\left[\log p_{\theta }\left(x|z\right)\right]-KL\left(q_{\phi }\left(z|x\right)||p\left(z\right)\right),$$

which can be optimized via Monte-Carlo Sampling [44].

## III. PROPOSED METHODS

In this section, we propose a novel deep mil model with ood capabilities named vaeabmil, built upon a vae, described in Section II-C and the attention mechanism described in Section II-B. The use of a vae is motivated by the need to model the data distribution in order to detect possible ood bags that may appear in the test set. The attention mechanism in abmil is used since it is the base of current sota mil models. In vaeabmil, instead of using the deterministic latent embedding z (with no ood capabilities) used in abmil, we make use of a vae which will replace the mil feature refiner $g_{\eta }$ and will be equipped with ood capabilities. Notice that this is a main novelty and an important benefit of vaeabmil: it is a deep mil model capable of both classifying bags and also detecting ood samples. For the embedded vae, we use the typical Gaussian observation and prior models presented in Section II-C, which will allow us to define a probabilistic ood score (see Section III-C). Let us now provide the mathematical formulation.

**FIGURE 1.**
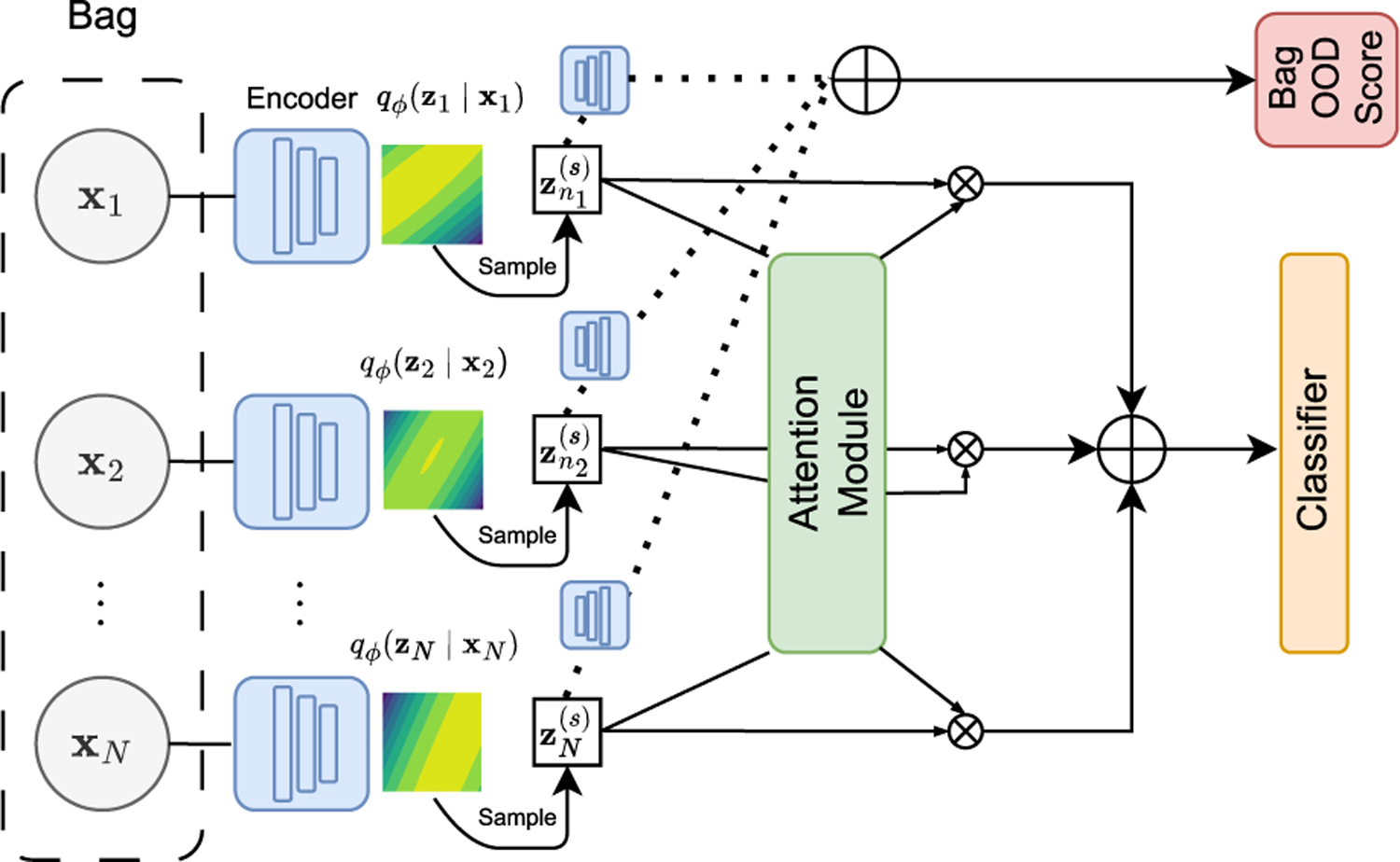
Graphical overview of the structure of vaeabmil. Each instance $x_{i}$ is encoded to obtain its approximated posterior distribution $q\left(z_{i}|x_{i}\right)$ using the encoder of the vae. Then, a sample $z_{i}\left(s\right)∼q\left(z_{i}|x_{i}\right)$ is obtained, which is used both in the classification and ood detection tasks. The classification is done using the Attention MIL paradigm on the samples from the approximated posterior. The OOD detection is performed using the decoder of the VAE.

**FIGURE 2.**
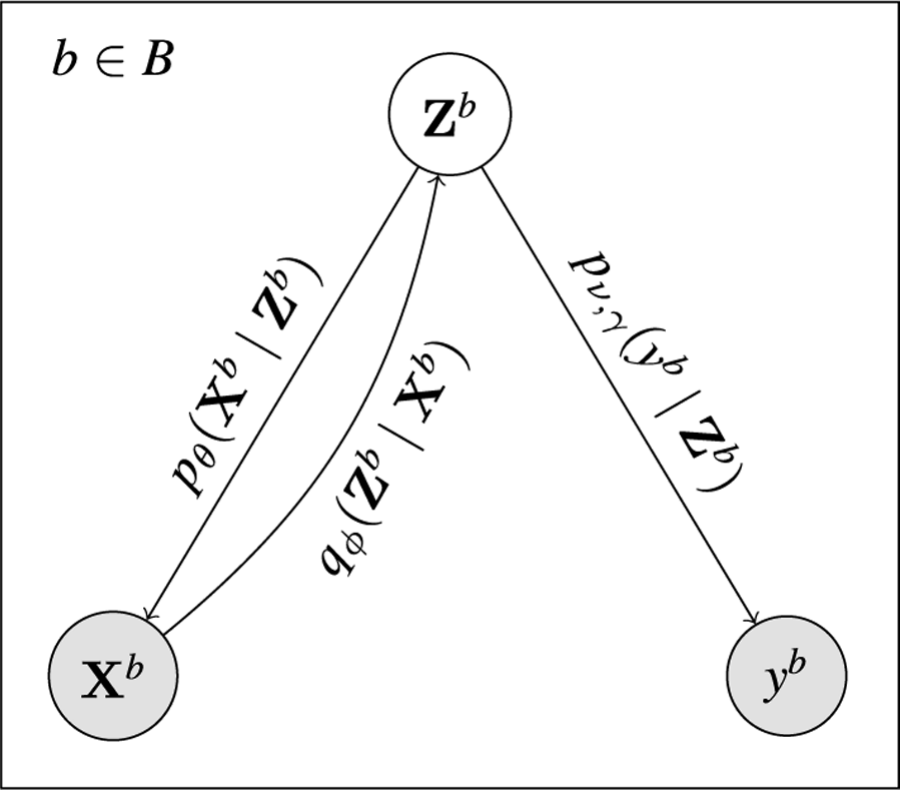
Probabilistic graphical depiction of vaeabmil. Given the latent variables $Z^{b}$, the bag label $y^{b}$ is independent of the observed bag ${\text{X}}^{b}$. We use $q_{\phi }\left(Z^{b}|X^{b}\right)$ for both the classification and ood detection tasks.

Given an observed bag $X^{b}$ and denoting by $Z^{b}$ the associated bag of *random* latent representations of its instances, each $z_{i}^{b}$ is responsible for the probabilistic generation of $x_{i}^{b}$, $i=1,\;\ldots ,\;N_{b}$ using the vae formulation described in Section II-C. We then make $Z^{b}$ solely responsible for the MIL classification of the bag, that is, $X^{b}$ and $y^{b}$ are conditionally independent given $Z^{b}$. We further use the attention mechanism in Equation (2) and the weighted-by-attention average of the instances in Equation (3) to obtain a bag representation $B^{b}$ that summarizes the information of the instances. Using the bag representation, we can compute the probability of the bag label $p_{\nu ,\gamma }\left(y^{b}|Z^{b}\right)=\text{Bern}\left(c_{\gamma }\left(B^{b}\right)\right)$, with $\nu =\left\{W,w\right\}$. A complete overview of the model can be observed in Figure 1. Also, the corresponding probabilistic graphical model is displayed in Figure 2. Letting $X=\left\{X^{1},\;\cdots ,X^{B}\right\}$, $Y=\left\{y^{1},\;\cdots ,y^{B}\right\}$ and $\mathbb{Z}=\left\{Z^{1},\;\cdots ,Z^{B}\right\}$ the joint distribution takes the form:

(6) $$\begin{array}{l} p_{\theta ,\nu ,\gamma }\left(Y,X,\mathbb{Z}\right)=p_{\nu ,\gamma }\left(Y|\mathbb{Z}\right)p_{\theta }\left(X|\mathbb{Z}\right)p\left(\mathbb{Z}\right)\\ =\prod_{b=1}^{B}\left(\underset{\text{Classification}\;\text{likelihood}}{\underset{︸}{p_{\nu ,\gamma }\left(y^{b}|Z^{b}\right)}}\prod_{i=1}^{N_{b}}\left(\underset{\text{VAE}\;\text{likelihood}}{\underset{︸}{p_{\theta }\left(x_{i}^{b}|z_{i}^{b}\right)p\left(z_{i}^{b}\right)}}\right)\right), \end{array}$$

where we have used the assumption of bag-level factorization in the classification-likelihood term and the instance-level factorization in the vae-likelihood term. Notice that, for each b, $Y^{b}$ and $X^{b}$ are independent given $Z^{b}$ but they become dependent when $Z^{b}$ is integrated on. This makes the unsupervised representation of the patches and the MIL classification dependent tasks. Note that, by removing the randomness on $Z^{b}$ and ignoring the decoder, we obtain the standard abmil. To make predictions, the latent variables $Z^{b}$ are marginalized using the posterior distribution $p\left(Z^{b}|X^{b}\right)$. Unfortunately, this distribution cannot be calculated in closed form and so we follow the procedure in vaes, resorting to a variational approximation that factorizes across bags and instances. This variational posterior distribution takes the form:

(7) $$\begin{array}{c} q_{\phi }\left(\mathbb{Z}|X\right)=\prod_{b=1}^{B}q_{\phi }\left(Z^{b}|X^{b}\right)=\prod_{b=1}^{B}\prod_{i=1}^{N_{b}}q_{\phi }\left(z_{i}^{b}|x_{i}^{b}\right)\\ \;\;\;=\prod_{b=1}^{B}\prod_{i=1}^{N_{b}}N\left(z_{i}^{b}|m_{\phi }\left(x_{i}^{b}\right),\sigma_{\phi }^{2}\left(x_{i}^{b}\right)I\right), \end{array}$$

where each $m_{\phi }$ and $\sigma_{\phi }^{2}$ are the ones defined for the vae (see Section II-C). Notice the simplification in the isotropic structure of the posterior covariance approximation for computational reasons, since the covariance matrix size scales quadratically with the number of instances in a bag, which can be very large depending on the patch and wsi sizes. Using more complex posteriors would drastically increase the optimization complexity of the model. We optimize the parameters of our model, ϕ, θ, ν, γ, by maximizing the elbo (or, equivalently, minimizing the minus elbo), which in the proposed model takes the form:

(8) $$L_{\phi ,\theta ,\nu ,\gamma }^{\text{VAEABMIL}}\left(X,\;Y\right)=E_{q_{\phi }\left(\mathbb{Z}|X\right)}\left[\log\frac{p_{\theta ,\nu ,\gamma }\left(Y,X,\mathbb{Z}\right)}{q_{\phi }\left(\mathbb{Z}|X\right)}\right]\;=E_{q_{\phi }\left(\mathbb{Z}|X\right)}$$

(9) $$\left[\log\frac{\prod_{b=1}^{B}\left(p_{\nu ,\gamma }\left(y^{b}|Z^{b}\right)\prod_{i=1}^{N_{b}}\left(p_{\theta }\left(x_{i}^{b}|z_{i}^{b}\right)p\left(z_{i}^{b}\right)\right)\right)}{\prod_{b=1}^{B}\prod_{i=1}^{N_{b}}q_{\phi }\left(z_{i}^{b}|x_{i}^{b}\right)}\right]\;=\sum_{b=1}^{B}E_{q_{\phi }\left(Z^{b}|X^{b}\right)}\left[\log p_{\nu ,\gamma }\left(y^{b}|Z^{b}\right)\right]$$

(10) $$+\sum_{i=1}^{N_{b}}E_{q_{\phi }\left(z_{i}^{b}|x_{i}^{b}\right)}\left[\log p_{\theta }\left(x_{i}^{b}|z_{i}^{b}\right)\right]$$

(11) $$-\sum_{i=1}^{N_{b}}KL\left(q_{\phi }\left(z_{i}^{b}|x_{i}^{b}\right)||p\left(z_{i}^{b}\right)\right).$$

The term in (9) is the classification log likelihood, which explains how well the model classifies the bags. The vae log likelihood, Equation (10), measures the quality of the instance reconstruction of the vae. The last term, (11) is the Kullback-Leibler (KL) divergence between the variational posterior and the Gaussian prior, which aims to regularize the variational posterior. The last two terms together are responsible for the ood detection and the learning of the manifold of the ind data. Notice that the KL divergence is crucial to maintain the properties of the latent space [46], therefore no term can be suppressed from this loss in order to maintain the performance of the model in both classification and ood detection tasks.

### A DETERMINISTIC VERSION OF vaeabmil

A.

Although the presented probabilistic model vaeabmil is theoretically sound, it is known that probabilistic models are harder to optimize than deterministic ones. This provides the motivation to derive daeabmil, a deterministic version of vaeabmil. To achieve this, we restrict the posterior distribution of vaeabmil in Equation (4) to be a Dirac’s delta $\delta \left(z-m_{\phi }\left(x\right)\right)$. Then, the instance latent representations Z become unique, rather than random variables. The loss function for daeabmil then becomes:

(12) $$L_{\phi ,\theta ,\nu ,\gamma }^{\text{DAEABMIL}}\left(X,\;Y\right)=\sum_{b=1}^{B}(\mu \log p_{\nu ,\gamma }\left(y^{b}|Z^{b}\right)\;\;\;\;\;\;\;\;\;\;\;\;\;\;\;\;\;\;\;\;+\alpha \;\sum_{i}^{N_{b}}{\left\|x_{i}^{b}-m_{\theta }\left(z_{i}^{b}\right)\right\|}^{2}+\beta \;\sum_{i}^{N_{b}}{\left\|z_{i}^{b}\right\|}^{2},$$

where $m_{\theta }\left(z_{i}^{b}\right)$ is the decoding of $z_{i}^{b}$ and μ, α, β are positive and add up to one. What is more, this model generalizes abmil, since taking α=β=0 abmil is recovered. Notice here, as we did with vaeabmil, that the last two terms together are responsible for the ood detection and the learning of the manifold of the training ind data. This manifold is now deterministic. With daeabmil we obtain faster inference, but it loses the probabilistic prediction.

### B. ind CLASSIFICATION PREDICTIONS

In vaeabmil, to make classification predictions on new test bags, we use the latent variables generated by the vae. Given a test bag $X^{★}=\left[x_{1},\;\cdots ,\;x_{N_{★}}\right]$ with $N_{★}$ instances, we define $Z_{\left(s\right)}^{★}=\left[z_{i}^{★,\left(s\right)},\;\cdots ,z_{N_{★}}^{★,\left(s\right)}\right]$, where $z_{i}^{★,\left(s\right)}\;∼\;q_{\phi }\left(z_{i}^{★}|x_{i}^{★}\right)$ is a sample from the approximated posterior of instance $x_{i}^{★}$. Then, we approximate the predictive distribution using S Monte Carlo samples as:

(13) $$p_{\nu ,\gamma }\left(y^{★}|X^{★}\right)=\int p_{\nu ,\gamma }\left(y^{★}|Z^{★}\right)p\left(Z^{★}|X^{★}\right)dZ^{★}\;\;\;\;\;\;\;\;\;\;\;\;\;=\int p_{\nu ,\gamma }\left(y^{★}|Z^{★}\right)q_{\iota }\left(Z^{★}|X^{★}\right)dZ^{★}\;\;\;\;\;\;\;\;\;\;\;\;\;\approx \;\frac{1}{S}\sum_{s}p_{\nu ,\gamma }\left(y^{★}|Z_{\left(s\right)}^{★}\right),$$

where, in the first equality, we have used the conditional independence of $y^{★}$ and $X^{★}$ given $Z^{★}$.

In the case of daeabmil, the instance embeddings are deterministic, so classification predictions are obtained as in (see Section II-B), with the significative difference that the latent embedding space was also trained to be robust to instance-level reconstruction (and, thus, capable to detect ood samples) using a deterministic autoencoder.

### C. OUT-OF-DISTRIBUTION DETECTION

One of the most important advantages of vaeabmil and daeabmil is their capability to model the instance-level data distribution $p\left(x\right)$ and, hence, detect ood bag samples. In this work, we propose to use an aggregation of the instance-level log marginal likelihood as the bag-level ood score. This score is motivated by the probabilistic meaning of the marginal likelihood: the lower marginal likelihood of x, the higher probability of x being ood.

To calculate this score, for each instance $x_{i}^{★}$ we first consider $z_{i}^{★}$, the unsupervised random representation of $x_{i}^{★}$, to compute the marginal distribution $p\left(x_{i}^{★}\right)$ which can be obtained using importance sampling with S Monte Carlo samples as:

(14) $$p\left(x_{i}^{★}\right)=\int \frac{p_{\theta }\left(x_{i}^{★}|z_{i}^{★}\right)p\left(z_{i}^{★}\right)}{q_{\phi }\left(z_{i}^{★}|x_{i}^{★}\right)}q_{\phi }\left(z_{i}^{★}|x_{i}^{★}\right)dz_{i}^{★}\;\;\;\;\;\;\;\;=E_{q_{\phi }\left(z_{i}^{★}|x_{i}^{★}\right)}\left[\frac{p_{\theta }\left(x_{i}^{★}|z_{i}^{★}\right)p\left(z_{i}^{★}\right)}{q_{\phi }\left(z_{i}^{★}|x_{i}^{★}\right)}\right]\;\;\;\;\;\;\;\;\approx \;\frac{1}{S}\sum_{s=1}^{S}\frac{p_{\theta }\left(x_{i}^{★}|z_{i}^{★,\left(s\right)}\right)p\left(z_{i}^{★,\left(s\right)}\right)}{q_{\phi }\left(z_{i}^{★,\left(s\right)}|x_{i}^{★}\right)}$$

This marginal distribution indicates how likely it is that a sample belongs to the training data distribution. We aggregate the instance-level score using the mean to compute vaeabmil’s bag-level ood score as:

(15) $$\text{LOGPX}\left(X^{★}\right)\;:=\frac{1}{N_{★}}\sum_{i=1}^{N_{★}}\left\{-\log p\left(x_{i}^{★}\right)\right\}.$$

The higher the logpx score, the more likely the bag is ood. Algorithmically, given a test bag $X^{★}$, the posterior distribution $q_{\phi }\left(z_{i}^{★}|x_{i}^{★}\right)$ of each of its instances is computed using Equation (4). Then, we sample S times from the approximated posterior of each instance, obtaining $z_{i}^{★,\left(s\right)}$ for $i=1,\;\ldots ,\;N_{★}$ and $\left(s\right)=1,\;\ldots ,\;S$. We then use Equation (14) to obtain an approximation of the marginal likelihood of each instance. Lastly the instance-level scores are aggregated using Equation (15). As a note, other aggregations (such as the maximum of the minus log marginal likelihoods) could be considered, but we have found the mean to be the best in practice (see the results with the max aggregation in Appendix B).

In the case of daeabmil, we can not compute the log marginal likelihood since the model is no longer probabilistic. However, we can compare the reconstruction with the original sample to see if the deterministic autoencoder can accurately reconstruct it. Since we expect ood samples to have higher reconstruction errors, we propose to use the mean of the reconstruction errors as daeabmil’s bag-level ood score:

(16) $$\text{RECERR}\left(X^{★}\right)\;:=\frac{1}{N_{★}}\sum_{i=1}^{N_{★}}{\left\|x_{i}^{★}-m_{\phi }\left(z_{i}^{★}\right)\right\|}^{2}.$$

As in the previous case, higher reconstruction errors indicate a higher chance of a sample being ood. Algorithmically, given a test bag $X^{★}$ we first compute its deterministic latent representation $Z^{★}$. Then, we reconstruct each instance using the decoder $m_{\theta }\left(x\right)$ and the bag-level ood score is obtained using Equation (16).

Interestingly, we have provided our model with prediction and ood detection capabilities. We have constrained the latent representations to be useful for the classification task but also for the ood detection task. The defined ood scores reflect the probability that an input belongs to the training distribution. Thus, they provide reliable and interpretable, tailored to the data, ood scores in mil settings. Recall that existing mil models must rely on model-agnostic ood scores “metrics derived from the model’s output rather than the underlying data distribution” to estimate the likelihood of an input being ood.

## IV. EXPERIMENTS

In this section we first describe the datasets, the experimental methodology, and the models used. This description is followed by the results supported by figures and graphical tables. A discussion of the limitations of the proposed approach concludes the section.

### A. DATASETS

Four different datasets are used to validate our proposed approach. The Camelyon16 (cam16) dataset [47] is used to address the task of detecting metastases in hematoxylin and eosin (H&E) stained wsis of breast cancer metastases. It is composed of 270 training and 130 test images. This dataset is public and it was presented in the Camelyon16 Grand Challenge.^1^

The Panda (panda) dataset [48] is a public dataset that was presented in the Panda Grand Challenge^2^. It contains prostate tissue wsis. Here we use it for a binary cancer-no cancer classification problem. In total, panda contains 8822 training slides and 1794 test slides.

Studies of sentinel lymph node biopsies for breast cancer show that 1.6% contain lymphoma. The third dataset (bcell) contain 26 lymph node tissue wsis diagnosed with diffuse large B-cell lymphoma. Here it will be used as ood data.

The fourth dataset (artif) contains 27 prostate tissue slides with different types of artifacts such as blur, foreign tissue or technical artifacts. Here it will be used as ood data. This is a very interesting challenge since artifacts commonly appear in real-world clinical scenarios.

The datasets have been carefully selected to offer a great variability of scenarios. Two different main tissue types are used for training (breast cam16 and prostate in panda). Those datasets also present differences in the size of their wsis and, hence, in the average number of extracted patches per slide. In the experiments bcell will be used as ood for cam16 since they both contain lymph node tissue. artif will be used as ood for panda. Both contain prostate wsis.

The four datasets are processed as follows. For each image, 512 × 512 pixel patches (instance) are extracted with the highest available resolution. The provided masks in cam16 and panda are used to produce bag labels while instances remain unlabelled. Since prostate tissue biopsies (panda) are smaller than lymph node sections (cam16), panda bags contain on average a smaller number of instances. Patch features are then extracted utilizing two different pre-trained models: Resnet50 with Barlow Twins (BT) self-supervised learning [49], using the weights provided in [50], and the general-purpose self-supervised foundation model for pathology uni [51]. uni was trained using more than 100 million images across 20 major tissue types. The usage of these two feature extractors allows the analysis of their influence in the classification and ood detection tasks. Patches and feature extraction is performed using the code from clam [52].^3^

### B. EXPERIMENTAL DESIGN

In this work, we assume that detecting ood samples is a test-time task. That is, our training (ind) datasets will be free from ood data, and the ood samples will appear during testing. Thus, to evaluate each model, each experiment consists of two different steps:

1. Classification step, where each model is trained on ind datasets (cam16 and panda, independently), and evaluated in the ind test set.
2. ood detection step, where we use the already trained models to measure their ood detection performance using an ood dataset.

For the classification task, the proposed models are compared with the following five sota mil models: dtfdmil [15] which uses pseudo bags to create a double-tier mil with distilled bag features, transmil [12], which uses a Transformer architecture to create bag representations which take into account instance correlations, dsmil [14], which uses instance correlations adding a pyramidal fusion of wsi features, and clam [52], which uses multiple attention branches for each class. Lastly, we also use the baseline abmil [11].

**FIGURE 3.**
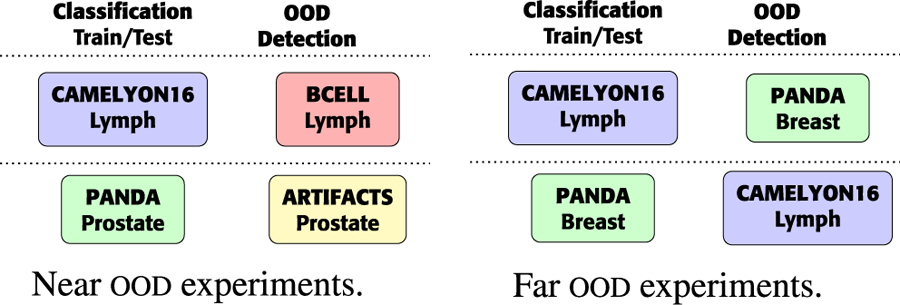
Graphical description of Near and Far OOD experiments. The main tissue type is indicated under the dataset name. Each experiment is performed using two feature extractors (UNI and BT).

In the ood detection task, we create four pairs of datasets (ind data, ood data): (cam16, bcell), (panda, artif), (cam16, panda), (panda, cam16). In the first two pairs, (cam16, bcell), (panda, artif), ind and ood slides share the main tissue. Therefore these experiments are defined as *Near OOD* detection scenarios, representing a harder ood task due to the similarity of the tissues present in the slides. The other two pairs are considered a *Far OOD* detection problem. A summary of these experiments can be found in Figure 3.

To perform the ood detection task, bag-level ood scores are computed. logpx (Equation (15)) and recerr (Equation (16)) are respectively proposed for vaeabmil and daeabmil. For the models we compare against, since they are not designed to handle ind/ood discrimination, we resort to post-hoc ood scores. Using the model logits ℓ, we compute the Maximum Logit Score (mls) [26] and the Entropy of the prediction [23], which (for a two class problem) takes the form

(17) $$H\left(p\right)=-\left(p\log p+\left(1-p\right)\log\left(1-p\right)\right),$$

with $p=\text{sigmoid}\left(\ell \right)$. Entropy and mls scores are also computed for vaeabmil and daeabmil. In this section, we report, for each model, the highest metric value obtained across all the ood scores. In Appendix B, we provide complete results for all models with the different ood scoring methods. Notice that other model-agnostic ood scores could be selected, but Entropy and mls are the most frequently used in the ood literature.

To compare the results, auc [53] is used. It quantifies a model’s ability to distinguish between positive and negative classes across all possible classification thresholds. In the classification task, model logits are used to compute the auc. In the ood detection task, the auc is computed based on the ood scores obtained for each model.

### C. IMPLEMENTATION DETAILS

Each model is run three times with different train/validation splits to provide statistically reliable results. We split 20% of the train set and used it as the validation set. We train each model for 100 epochs in cam16 and 50 in panda with no early stopping, using a learning rate of 10^−4^ for all the models but transmil, for which we use 10^−5^. For each run, test metrics are computed using the model weights corresponding to the highest validation auc achieved during training. We code the models using *Pytorch* [54], and we use the Adam optimizer [55].

For the architecture of vaeabmil and daeabmil, in both cases we use simple autoencoders composed of three linear layers with sizes [512, 256, 128] as the encoder and we utilize the same dimensions for the decoder. In daeabmil, we use μ=1, and α=β=0.3 in Eq. (12) to train the model. To predict the variances in vaeabmil, we produce a single value that is used across all the latent dimensions and use S=1 Monte Carlo sample for inference. The models are trained in a single Nvidia 3090 GPU with 24 gigabytes of RAM. The rest of the model follows the implementation of the original abmil. The code is available at https://github.com/fjsaezm/VAEABMIL.

### D. CLASSIFICATION RESULTS

**FIGURE 4.**
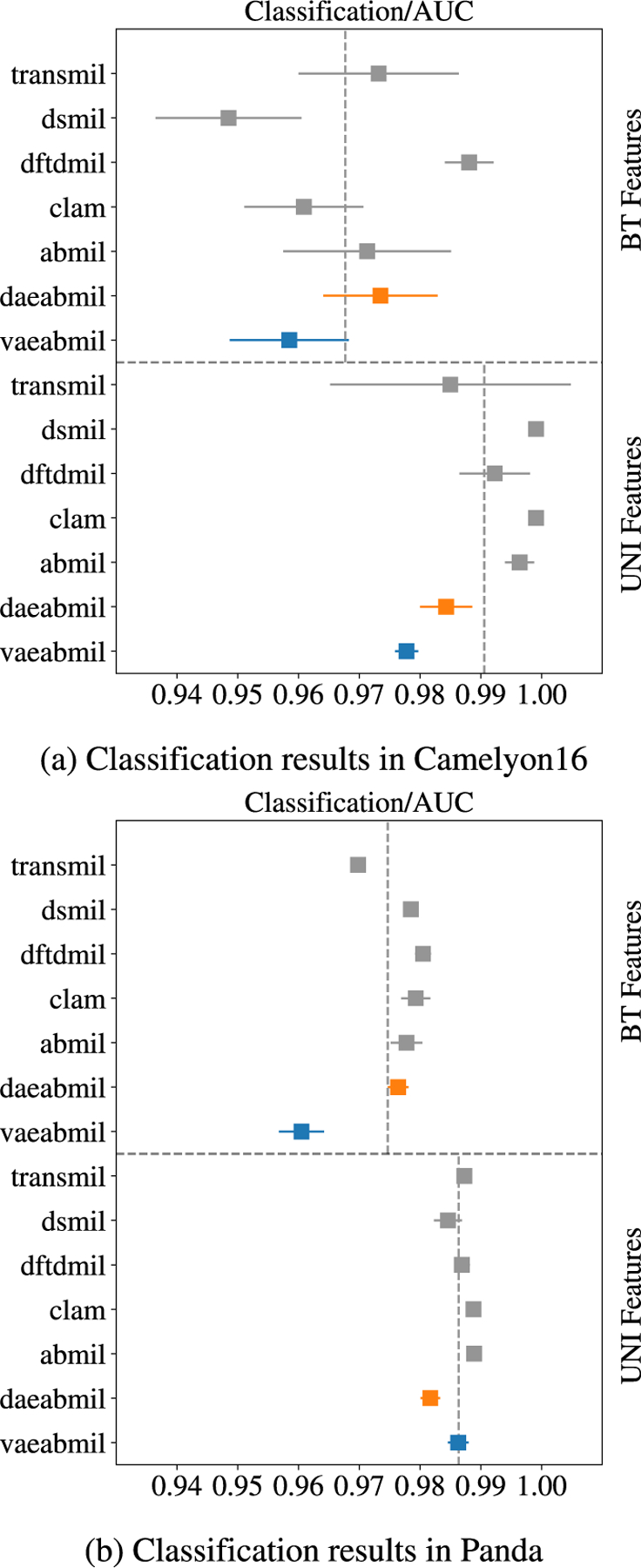
Classification results for both cam16 and panda datasets. The presented metric is the test auc(right is better). Mean and standard deviations are reported for each model. The results with both feature extractors are separated by the horizontal dashed line. The vertical, dashed lines represent the mean performance of the models using each feature extractor.

Figure 4 shows the auc metric in the bag classification task for cam16 and panda, using both bt and uni feature extractors.

For the cam16 dataset, Figure 4a shows that the models are, regardless the feature extractor, very accurate for this benign/malignant classification, with the worst performance being better than 0.95 auc. With both feature extractors, vaeabmil and daeabmil perform similarly to the rest of the models. In the cases where our models perform worse than the rest, the highest difference in auc does not exceed 1%. This is compensated by their additional ood detection capabilities. Comparing the results across the different feature extractors, the models perform clearly better when using UNI. This is observed in the vertical dashed black lines, which represent the average of the means of all the models using the corresponding features. This indicates that uni produces excellent features of the patches, facilitating the classification task.

Figure 4b presents the classification results on the panda dataset, which show trends similar to those observed in cam16. When using bt features, vaeabmil performs approximately 2% worse than the other models, whereas daeabmil performs comparably to the sota methods. This 2% performance gap between vaeabmil and daeabmil is also observed in cam16, highlighting the optimization advantages of daeabmil over vaeabmil for classification tasks using bt features. In contrast, when using uni features, all models achieve near-perfect classification performance, with auc scores exceeding 0.98.

**FIGURE 5.**
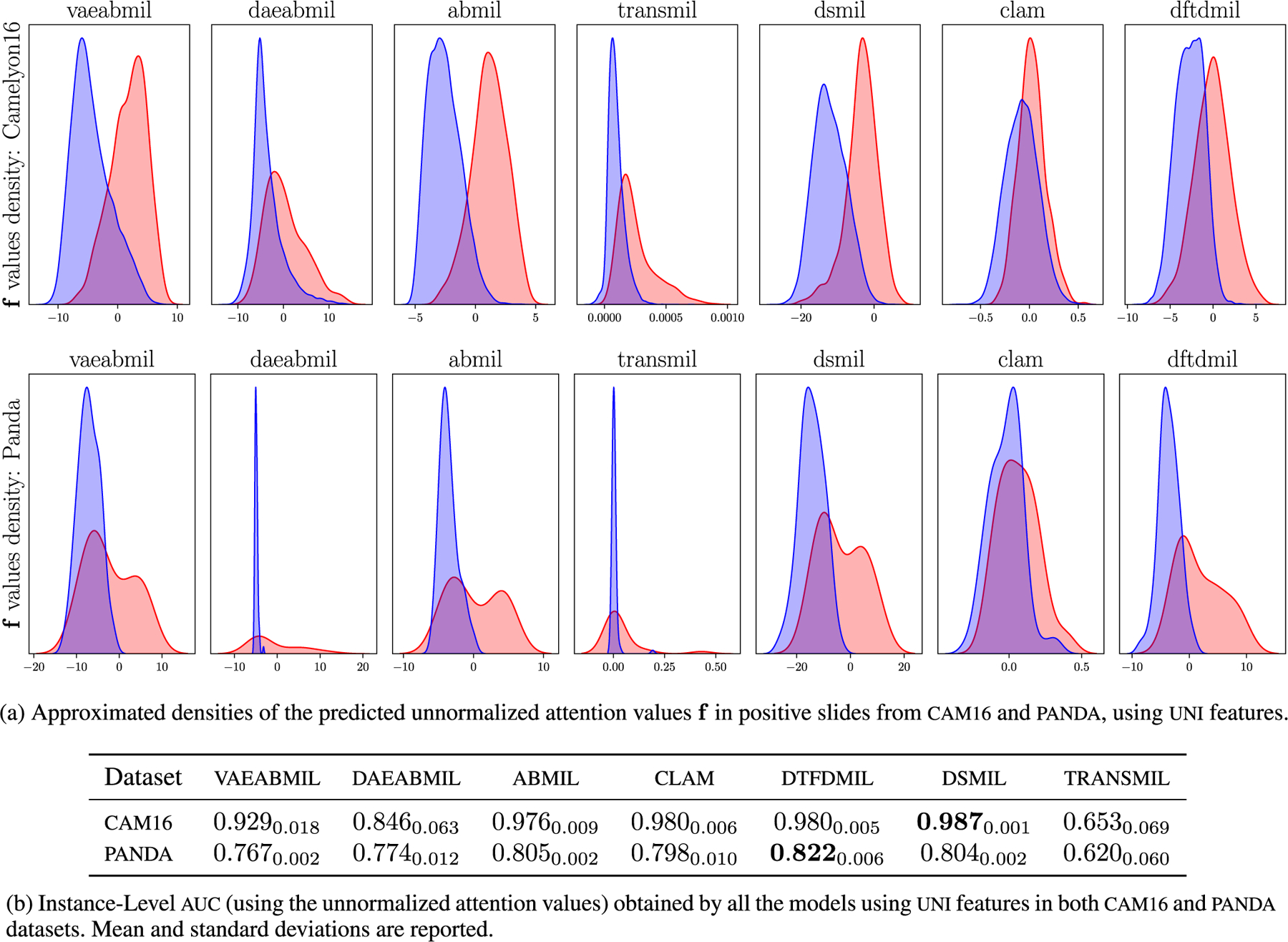
Instance-level results, using the unnormalized attention values f and uni features.

To conclude this section, we compare the attention values (Equation (2)), provided by each classifier. Figure 5a shows the instance-level attention prediction in positive bags in both cam16 and panda, using uni features. Visually, vaeabmil performs better than daeabmil in cam16 and equally in panda, emphasizing the benefits of obtaining a probabilistic, continuous latent space. This is confirmed by the quantitative results shown in Table 5b. Compared with the rest of the models (except for transmil), we observe that the proposed models perform slightly worse. However, as it was shown in Figure 4, the bag-level performance of our methods is similar to that of the rest of the models. Notice that, similarly, transmil obtains poor attention values but high bag-level classification metrics.

### E. FAR ood DETECTION

ood detection results are now presented, starting with Far ood experiments, where ind and ood data do not share the main tissue type and, therefore, we expect an easier task. Results shown in this section are supported by the statistical significance analysis performed in Appendix A.

#### 1) (cam16, panda)

**FIGURE 6.**
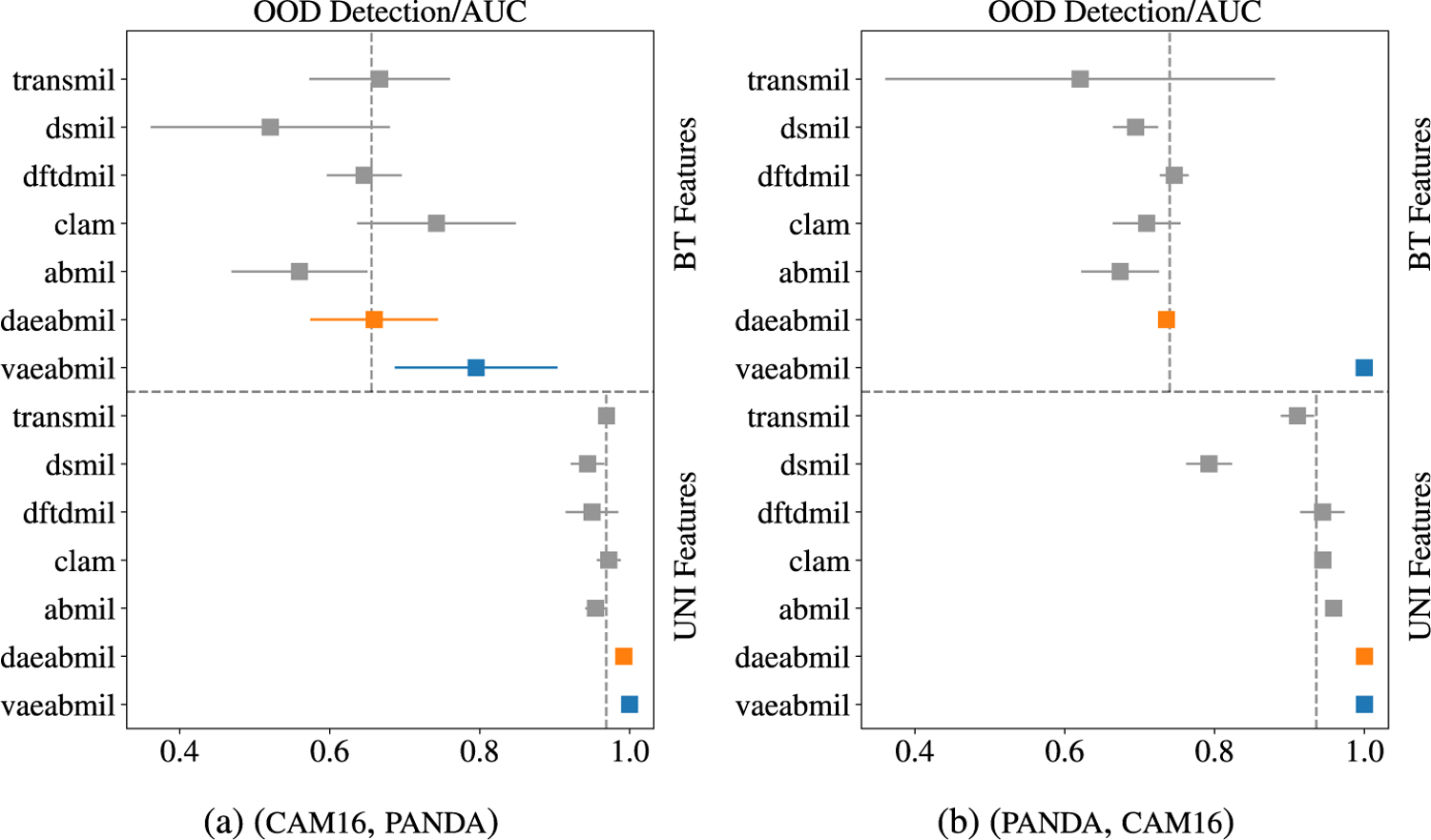
Far ood detection results. The presented metric is the auc(right is better). Mean and standard deviations (which are almost zero in some cases) are reported for each model. The results with both feature extractors are separated by the horizontal dashed line. The vertical, dashed lines represent the mean performance of the models using each feature extractor.

Models trained with cam16 (see the classification performance in Section IV-D) are now evaluated using panda as ood dataset. Figure 6a shows that, when bt features are used, vaeabmil obtains the best ood detection result, and daeabmil is on average with the rest of the models. Such behaviour is caused by two main reasons: a) The difficulties in the two-task optimization process which our proposal suffers from (specified in Section IV-G), and b) the deterministic latent space in daeabmil might not be flexible enough to produce far-apart representations for the cam16 and panda datasets. This highlights the benefits of the smooth, probabilistic latent space that the vae in vaeabmil produces. Also in Figure 6a, when using uni features, the ood detection performance of the rest of the models increases, due to the highly refined features that this foundation model produces. However, our models obtain the best result in ood detection due to their explicit data-distribution modelling capability.

**FIGURE 7.**
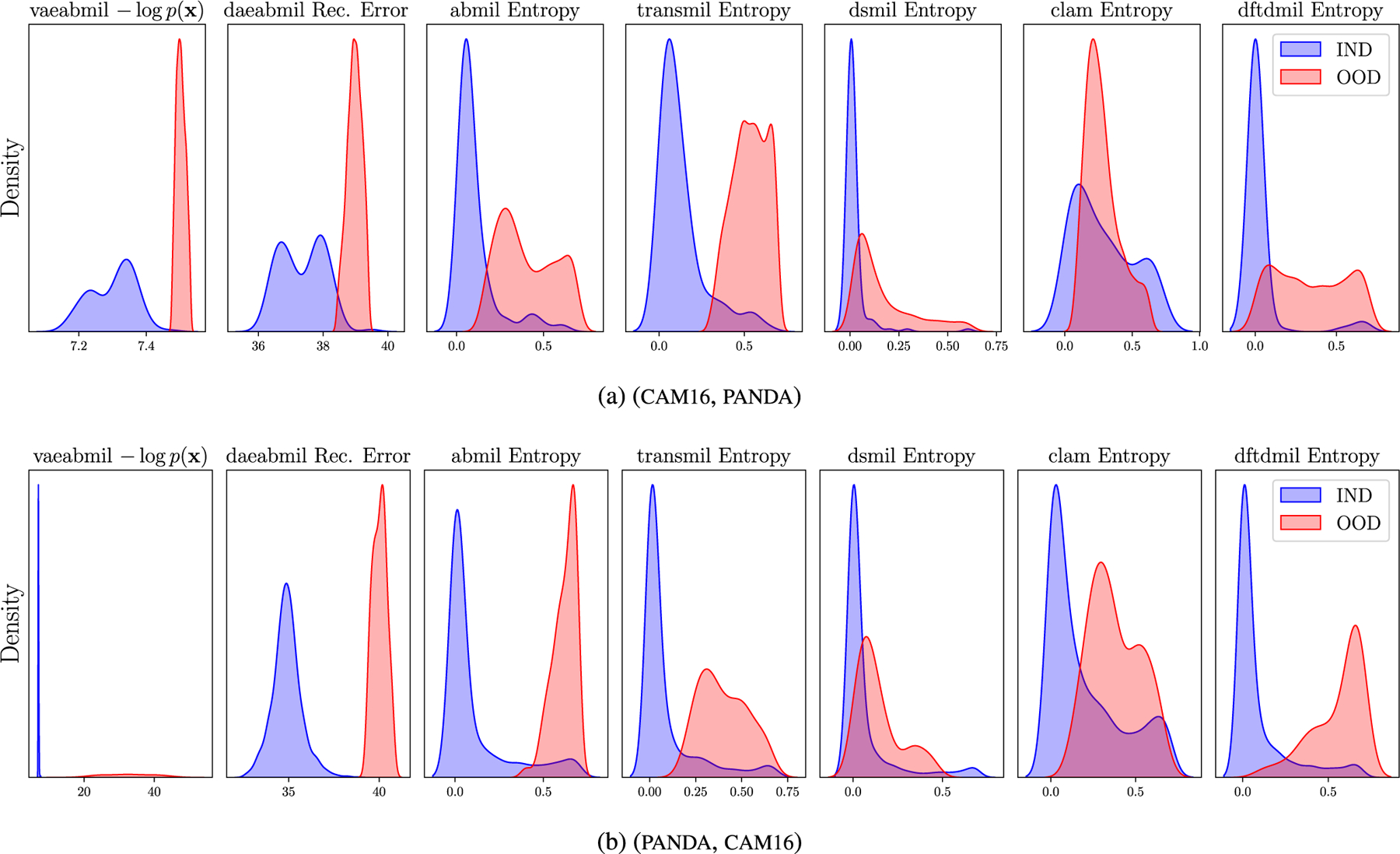
Approximated densities of the bag-level ood scores produced by the models in the Far ood detection experiments, using uni features.

**FIGURE 8.**
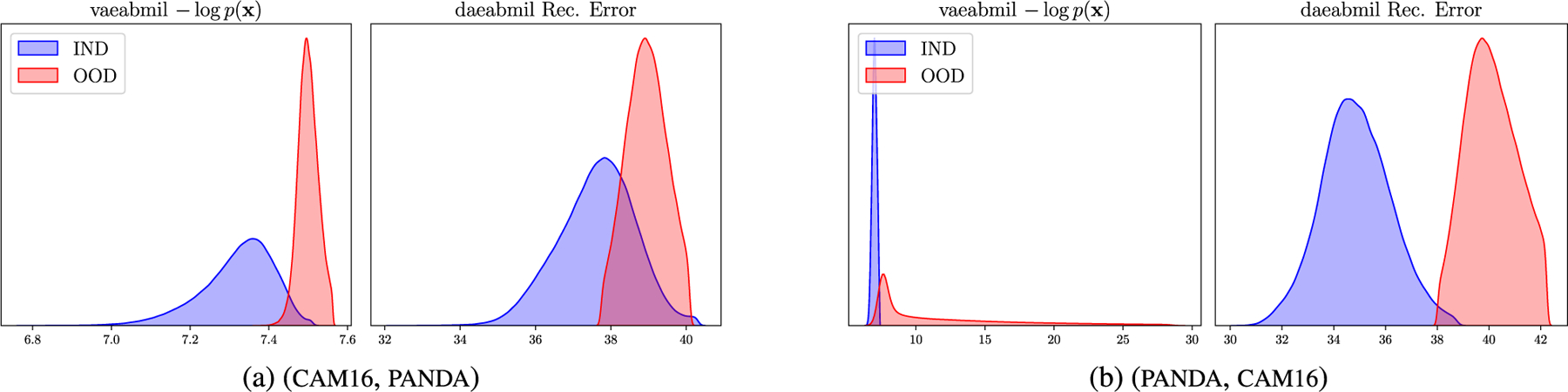
Approximated densities of the instance-level ood scores produced by vaeabmil and daeabmil in the Far ood detection experiments using uni features.

Figure 7a, shows the slide-level ood score for all the models using uni features. In this case, and although our proposals still perform better than the others, we observe an also good performance by transmil and abmil, which also produce two different distributions for ind and ood bags. Figure 8a shows the instance-level predicted ood scores by our models, using uni features.^4^ The separation that our models produce is large enough to clearly distinguish between ind and ood instances. This is coherent with the fact that the slides in this ood detection problem contain different types of tissue.

#### 2) (panda, cam16)

Now, we use panda ad ind dataset and cam16 as ood dataset. Figure 6b shows the ood detection results. The results are similar to those obtained in the previous experiment: we again observe that daeabmil performs on pair with the rest of the models. This supports our idea that the features produced by bt for panda and cam16 are not discriminative enough to differentiate them through a deterministic autoencoder which produces a non-continuous latent space. vaeabmil, however, obtains a perfect auc score, highlighting the benefits of using a continuous, probabilistic latent space and modelling the likelihood of the data to detect out of distribution samples. When using uni features, daeabmil and vaeabmil are capable to detect all ood bags correctly, outperforming the rest of the models.

Figure 7b depicts the bag-level ood scores obtained by all the models, showing that thanks to uni features, all the models separate the distributions of the ind and ood sets, with vaeabmil and daeabmil doing it perfectly. The good auc results are supported by the correct instance-level ood discrimination shown in Figure 8b, where in both cases we observe a instance-level separation between ind and ood scores.

### F. NEAR odd DETECTION

To end the experimental section, we present the Near ood detection problems where, as indicated in Section IV-B, the ind dataset and the ood dataset share the main tissue type. The results shown in this section are supported by the statistical significance analysis performed in AppendixA.

#### 1) (cam16, bcell)

**FIGURE 9.**
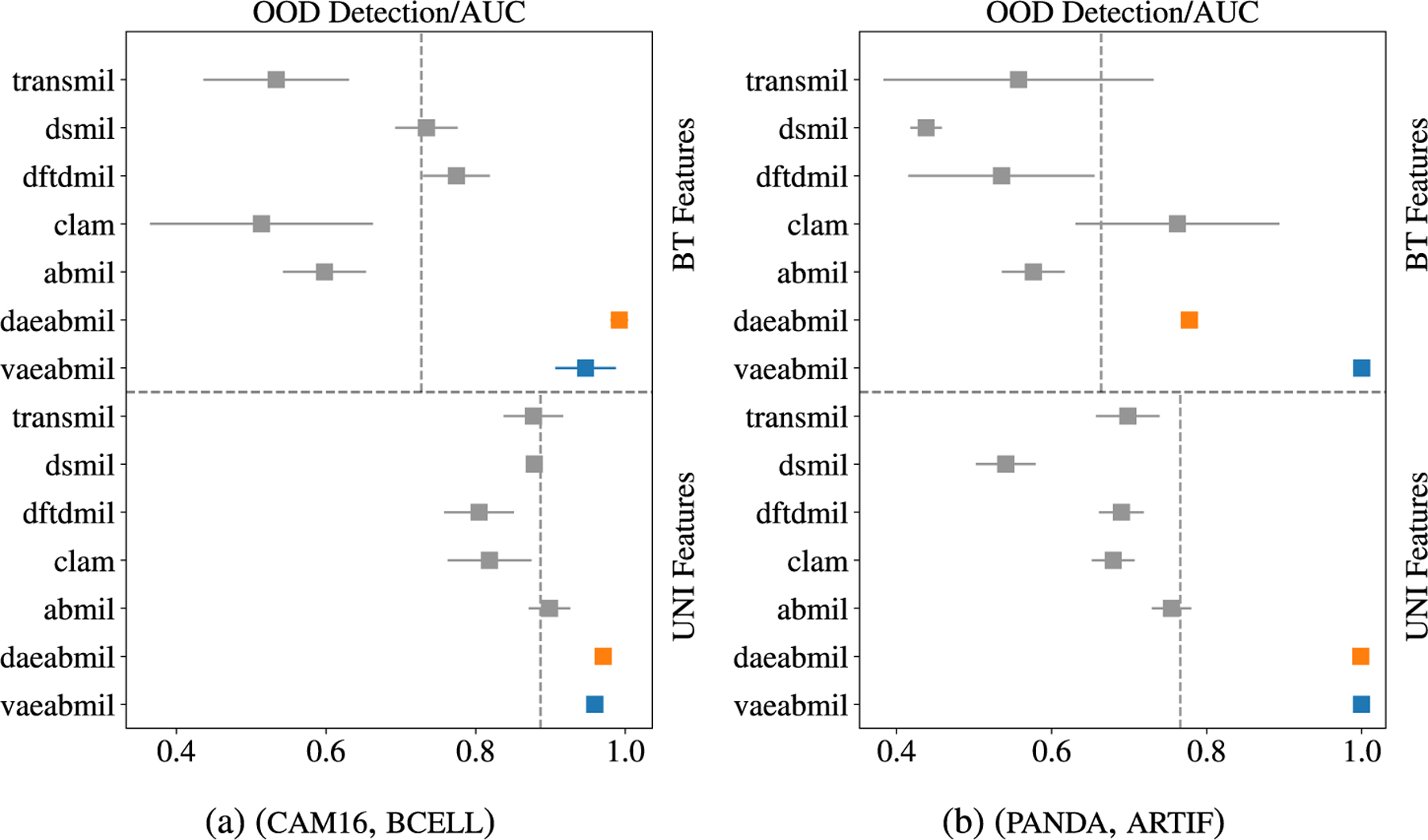
Near ood detection results. The presented metric is the auc(right is better). Mean and standard deviations (which are almost zero in some cases) are reported for each model. The results with both feature extractors are separated by the horizontal dashed line. The vertical, dashed lines represent the mean performance of the models using each feature extractor.

In this scenario, ind and ood wsis share the main tissue type but differ in their medical diagnosis. As described in Section IV-A, positive slides in cam16 present cancer metastasis in lymph node sections, while bcell wsis have been diagnosed with diffuse large B-cell lymphoma. This poses, a priori, a more difficult ood detection problem. Figure 9a shows, the ood detection results. Observing this Figure, we highlight that:

- vaeabmil and daeabmil excel at detecting ood samples, obtaining an almost perfect auc using any of the used features. This indicates that the autoencoders in both methods have learned to assign higher logpx and recerr, respectively, to ood samples than to ind ones.
- We observe considerably worse results for the rest of the models. When using bt features, the auc is approximately 0.6 in some cases, indicating that the entropy of the predictions is the same for both ind and ood data. This is an important problem with current sota mil models, since their predictions are not well calibrated and can not detect ood samples. This poses an important problem for their use in real diagnosis applications.
- When uni features are used, the rest of the models show a strong improvement in the ood detection, which correlates with the improvement in the classification auc. We state that the foundation model uni produces more discriminative features for the downstream tasks, separating ood instances further away from ind data.

**FIGURE 10.**
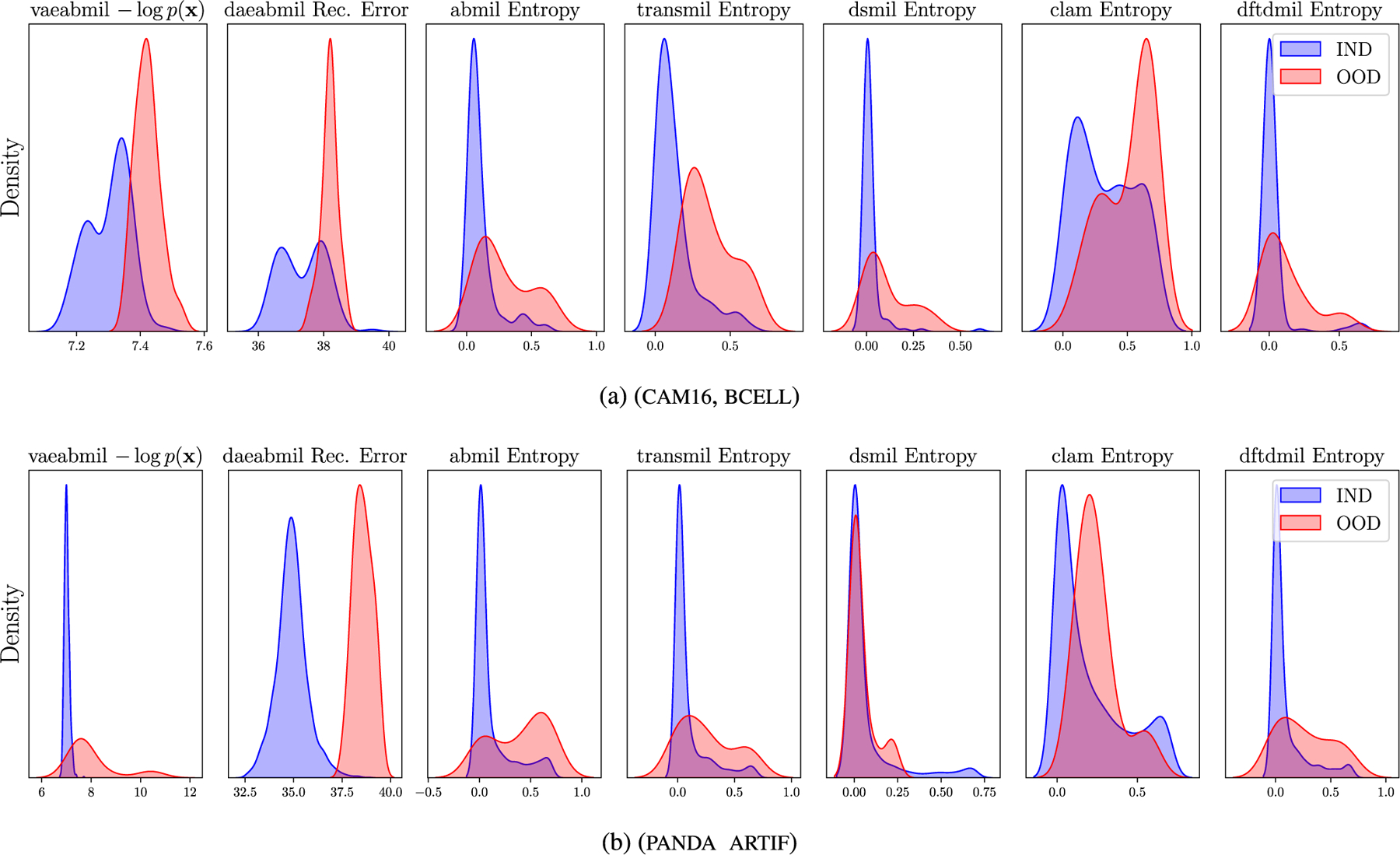
Approximated densities of the bag-level ood scores produced by the models in the Near ood detection experiments, using uni features.

**FIGURE 11.**
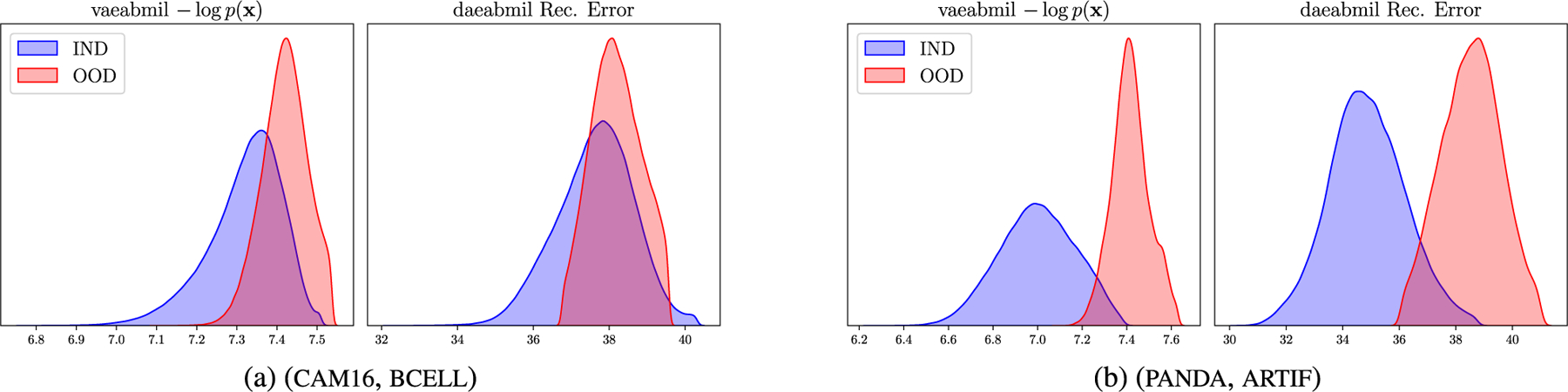
Approximated densities of the instance-level ood scores produced by vaeabmil and daeabmil in the Near ood detection experiments using uni features.

**FIGURE 12.**
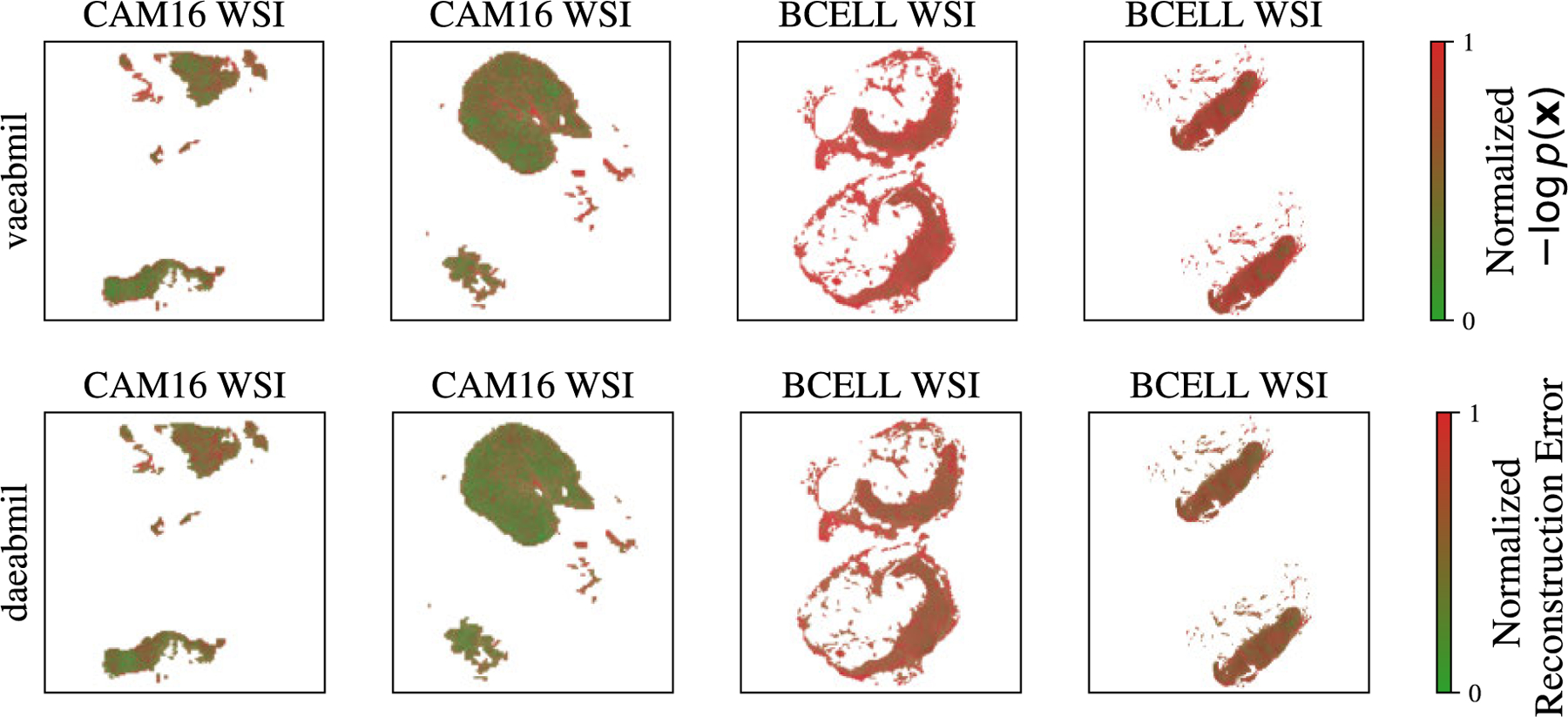
**Top row**: **− $\log\;p\left(x\right)$** values obtained by vaeabmil for each patch in both ind and ood wsis. **Bottom Row**: reconstruction error of each patch in both ind and ood wsis, obtained by daeabmil. In each rows, uni features are used, and the predicted instance-level values are jointly normalized along the wsis. vaeabmil and daeabmil assign similar instance-level ood scores in ind samples, being much higher in the ood dataset (bcell) than in the ind one (cam16).

Figure 10a displays the WSI-level ood score produced by each of the models. This figure reveals that the rest of the models assign very similar ood scores to ind and ood wsis, which is a key drawback when using those models in a real world scenario like the one we are presenting. Our models, in contrast, produce separated distributions that may alert the pathologist when diagnosing a patient. Although transmil and abmil may seem to differentiate between ind and ood distributions, the auc metric in Figure 9a reveals that their ood detection performance is still worse than vaeabmil and daeabmil. Figure 11a, shows the instance-level ood predictions of vaeabmil and daeabmil, using uni features. We observe that, even though there is overlapping between the estimated densities of the scores of ind and ood instances, there is a shift in the mean of the distributions of ind and ood instances, specially in vaeabmil. Such distribution shift is the cause of the remarkable ood detection capabilities of our models. Thanks to averaging the instance-level ood scores, ood bags are perfectly detected. Notice that the instance-level ood scores show which areas of the wsi are poorly reconstructed by the autoencoders and are, thus, more relevant to identify the slide as ood. We show an example of this behaviour in Figure 12, where higher instance-level recerr/logpx are obtained in bcell (ood) compared to the cam16 (ind) patches.

#### 2) (panda, artif)

In the last experiment, we assess the ood detection capabilities of our models in another real clinical scenario. Models trained in panda are evaluated by testing their ability to identify prostate slides containing pathologist-annotated artifacts. This represents a highly relevant case, as artifacts are commonly encountered in real-world wsis. Figure 9b shows that our models, specially vaeabmil, outperform the sota models in this task when using bt features. When using uni features, the difference between our proposals and the sota models also becomes clear for daeabmil, which highlights the importance of using a foundation model as feature extractor for ood detection tasks. This is also observed in the estimated densities of the bag-level ood scores shown in Figure 10b. The conclusions are the same as the ones presented in Section IV-F1, showing consistency of our method. These results are clear indicator of the benefits of our proposal: our models present a novel approach that can perform the classification task on pair with the sota models while clearly outperforming them in the ood detection task.

**FIGURE 13.**
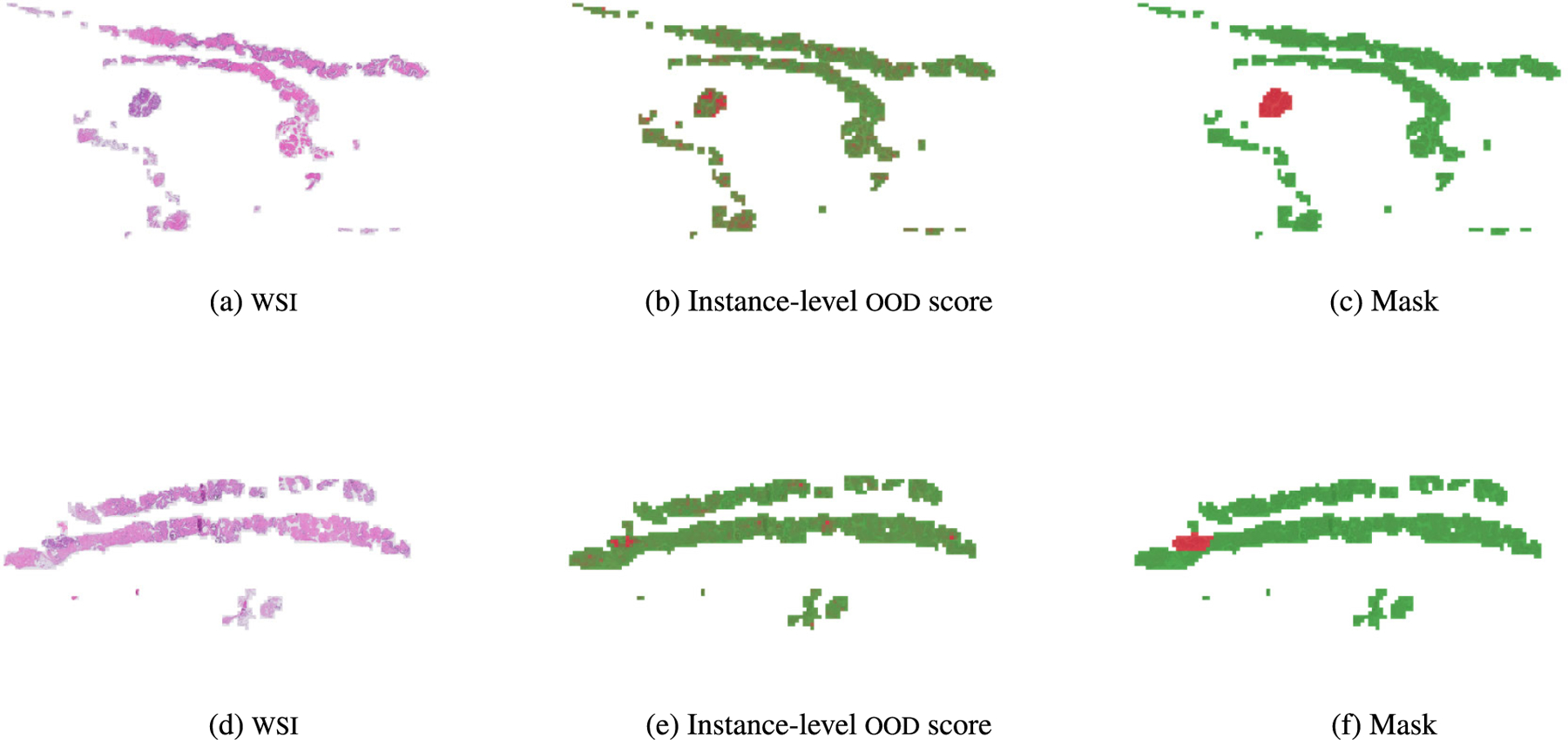
Visualization of two wsis from the artif dataset containing annotated artifacts. The corresponding instance-level ood scores predicted by vaeabmil and masks are shown. Each row corresponds to a different case. It is observed how vaeabmil assigns higher ood scores to the regions identified as artifacts in the mask.

Figure 11b shows overlapping between ind and ood instance-level distributions for this experiment. This is an expected behaviour since artif contains prostate tissue as panda does. However, thanks to aggregating the scores in the whole bags, artifact-containing bags are correctly identified as ood. Furthermore, in Figure 13 we leverage the instance-level ood score provided by vaeabmil to show that our proposal can be used to locate artifacts. This provides a visual tool for pathologists, adding to vaeabmil high value for clinical use.

### G. LIMITATIONS

**FIGURE 14.**
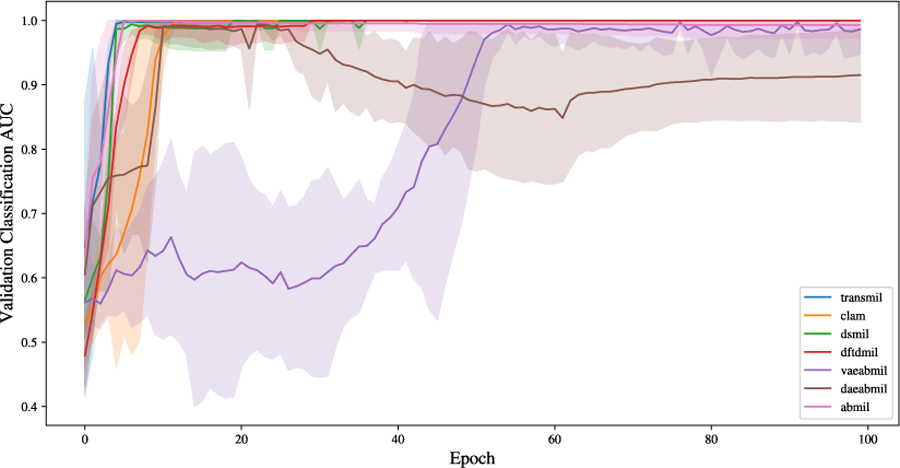
Validation auc for all the trained models in the cam16 dataset using features from UNI. Mean and 95% confidence intervals are shown per each model. The convergence of vaeabmil is slower than that of the rest of the models. Also, daeabmil shows a performance decrease due to the double-objective optimization task.

Both proposed models exhibit one main limitation when compared to the other deep mil models: our methods are harder to optimize than the rest. The reason for this is that, in both cases, the loss function to be optimized is composed of a classification-related term and two ood detection-related terms. Thus, jointly optimizing all the terms compromises the effectiveness of the model, specially in the classification task, as we have observed in the results. This can also be observed in Figure 14, where we plot the classification auc in the validation set during the optimization process in the cam16 dataset using uni features. We observe that vaeabmil converges slower than the rest of the models. daeabmil, converges as fast as clam, but lowers its performance as the training process advances due to the need to also optimize for instance-level reconstruction task.

Nevertheless, even with this limitation, our proposals obtain comparable classification results and better ood detection metrics, making them very useful in real-world scenarios.

## V. CONCLUSION AND FUTURE WORK

While the apparition of ood samples is very frequent in digital pathology, current mil sota methods are not designed to reliably quantify whether a test bag belongs to the training data distribution. This limitation poses a great risk of incorrect predictions when unexpected tissues are encountered in real-world clinical settings. With this motivation, we propose a novel probabilistic deep mil method with ood capabilities. Our model, vaeabmil, generalizes the well-known ABMIL using a vae to model the data distribution, which gives the mil method the ability to detect ood samples by aggregating the marginal likelihood of the instances as an ood score. Also, we have proposed a deterministic version, daeabmil, which leverages the reconstruction error as a deterministic ood score. The main novelty of the proposed models is that they are defined and trained to perform two different tasks (bag-level classification and ood detection) simultaneously, which none of the previous mil methods is doing.

Extensive experimentation shows that vaeabmil and daeabmil are competitive with the rest of the sota methods in the classification task. Furthermore, and very importantly for the design of CAD systems, they outperform current mil methods at detecting ood samples in both Near and Far ood scenarios. The experiments also highlight the importance of using a foundation model as a feature extractor.

This work opens several promising directions for future research. One possibility is to extend the use of a vae in combination with more complex mil methods such as transmil or dtfdmil. Another is to explore alternative generative models for learning the data distribution. Both approaches have the potential to enhance the ood detection performance of mil models.

## Figures and Tables

**FIGURE 15.**
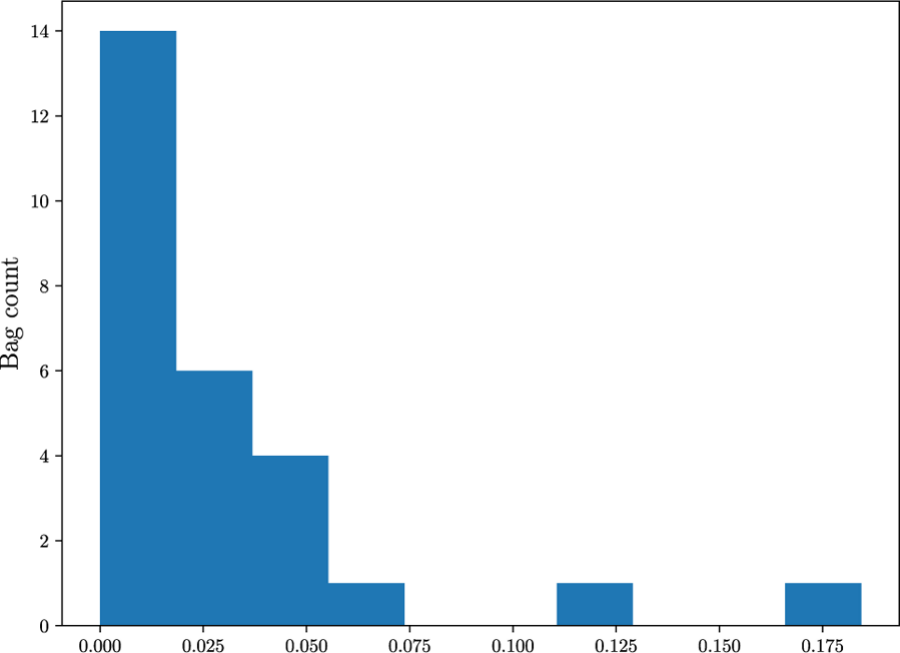
Histogram of the proportion of each wsi covered by an artifact in the artif dataset. The proportion does not exceed 17.5%.

**TABLE 1.** T-test results comparing the ood auc in the (cam16,panda) experiment using uni features.

(a) Statistical comparison for vaeabmil				
Model	OoD/auroc	t_stat	p_value	Significant
vaeabmil	0.9999 ± 0.0001	-	-	-
transmit	0.9690 ± 0.0059	8.9761	0.0122	True
clam	0.8300 ± 0.1985	1.4827	0.2764	False
dsmil	0.9438 ± 0.0224	4.3511	0.0490	True
dftdmil	0.9497 ± 0.0351	2.4722	0.1320	False
daeabmil	0.9923 ± 0.0001	352.6000	0.0000	True
abmil	0.9544 ± 0.0135	5.8111	0.0284	True
(b) Statistical comparison for daeabmil				
Model	OoD/auroc	t_stat	p_value	Significant

daeabmil	0.9923 ± 0.0001	-	-	-
transmil	0.9690 ± 0.0059	6.7671	0.0211	True
clam	0.8300 ± 0.1985	1.4161	0.2924	False
dsmil	0.9438 ± 0.0224	3.7590	0.0640	False
dftdmil	0.9497 ± 0.0351	2.0995	0.1706	False
vaeabmil	0.9999 ± 0.0001	−352.6000	0.0000	True
abmil	0.9544 ± 0.0135	4.8494	0.0400	True

**TABLE 2.** T-test results comparing the ood auc in the (cam16,bcell) experiment using uni features.

(a) Statistical comparison for vaeabmil				
Model	OoD/auroc	t_stat	p_value	Significant
vaeabmil	0.9592 ± 0.0030	-	-	-
transmil	0.8772 ± 0.0398	3.3333	0.0794	False
clam	0.8185 ± 0.0560	4.4629	0.0467	True
dsmil	0.8784 ± 0.0100	10.7940	0.0085	True
dftdmil	0.8045 ± 0.0466	5.7613	0.0288	True
daeabmil	0.9704 ± 0.0084	-1.7167	0.2282	False
abmil	0.8987 ± 0.0277	3.9603	0.0582	False
(b) Statistical comparison for daeabmil				
Model	OoD/auroc	t_stat	p_value	Significant

daeabmil	0.9704 ± 0.0084	-	-	-
transmil	0.8772 ± 0.0398	4.8397	0.0401	True
clam	0.8185 ± 0.0560	4.4932	0.0461	True
dsmil	0.8784 ± 0.0100	38.1552	0.0007	True
dftdmil	0.8045 ± 0.0466	6.2819	0.0244	True
vaeabmil	0.9592 ± 0.0030	1.7167	0.2282	False
abmil	0.8987 ± 0.0277	4.0565	0.0557	False

**TABLE 3.** T-test results comparing the ood auc in the (panda,cam16) experiment using uni features.

(a) Statistical comparison for vaeabmil				
Model	OoD/auroc	t_stat	p_value	Significant
vaeabmil	1.0000 ± 0.0000	-	-	-
daeabmil	1.0000 ± 0.0000	2.0000	0.1835	False
dsmil	0.7926 ± 0.0306	11.7303	0.0072	True
clam	0.6966 ± 0.0516	10.1895	0.0095	True
dftdmil	0.9436 ± 0.0298	3.2799	0.0817	False
transmit	0.9106 ± 0.0223	6.9357	0.0202	True
abmil	0.9590 ± 0.0069	10.3725	0.0092	True
(b) Statistical comparison for daeabmil				
Model	OoD/auroc	t_stat	p_value	Significant

daeabmil	1.0000 ± 0.0000	-	-	-
vaeabmil	1.0000 ± 0.0000	−2.0000	0.1835	False
dsmil	0.7926 ± 0.0306	11.7294	0.0072	True
clam	0.6966 ± 0.0516	10.1893	0.0095	True
dftdmil	0.9436 ± 0.0298	3.2798	0.0817	False
transmit	0.9106 ± 0.0223	6.9359	0.0202	True
abmil	0.9590 ± 0.0069	10.3694	0.0092	True

**TABLE 4.** T-test results comparing the ood auc in the (panda,artif) experiment using uni features.

(a) Statistical comparison for vaeabmil				
Model	OoD/auroc	t_stat	p_value	Significant
vaeabmil	0.9993 ± 0.0004	-	-	-
daeabmil	0.9988 ± 0.0001	2.2618	0.1521	False
dsmil	0.5408 ± 0.0386	20.3737	0.0024	True
clam	0.6794 ± 0.0276	20.3732	0.0024	True
dftdmil	0.6883 ± 0.0300	18.1359	0.0030	True
transmil	0.6982 ± 0.0410	12.8230	0.0060	True
abmil	0.7546 ± 0.0255	16.7655	0.0035	True
(b) Statistical comparison for daeabmil				
Model	OoD/auroc	t_stat	p_value	Significant

Model	OoD/auroc	t_stat	p_value	Significant
daeabmil	0.9988 ± 0.0001	-	-	-
vaeabmil	0.9993 ± 0.0004	-2.2618	0.1521	False
dsmil	0.5408 ± 0.0386	20.5614	0.0024	True
clam	0.6794 ± 0.0276	20.0429	0.0025	True
dftdmil	0.6883 ± 0.0300	17.9140	0.0031	True
transmil	0.6982 ± 0.0410	12.6833	0.0062	True
abmil	0.7546 ± 0.0255	16.5333	0.0036	True

**TABLE 5.** Tables with the ood detection results using multiple ood scores. mls stands for Maximum Logit score. In the scores defined for vaeabmil and daeabmil, max and mean indicate the Maximum aggregator and the Mean aggregator, respectively.

(a) ood detection results for the different ood scores in the (cam16, panda) experiment.						
Model	OoD/Entropy/auc	OoD/MLS/auc	OoD/LOGPXMAX/auc	OoD/LOGPXMEAN/auc	OoD/RECERRMAX/auc	OoD/RECERRMEAN/auc
abmil	0.954 ± 0.013	0.935 ± 0.031	-	.	-	-
clam	0.830 ±0.199	0.826 ± 0.203	-	-	-	-
daeabmil	0.963 ±0.011	0.968 ± 0.007	-	-	0.457 ± 0.031	0.992 ± 0.000
dftdmil	0.950 ± 0.035	0.961 ±0.015	-	-	-	-
dsmil	0.944 ± 0.022	0.923 ± 0.019	-	-	-	-
transmil	0.969 ± 0.006	0.960 ± 0.013	-	-	-	-
vaeabmil	0.979 ± 0.007	0.970 ±0.011	0.680 ± 0.109	1.000 ± 0.000	-	-
(b) ood detection results for the different ood scores in the (cam16, bcell) experiment.						
Model	OoD/Entropy/auc	OoD/MLS/auc	OoD/LOGPXMAX/auc	OoD/LOGPXMEAN/auc	OoD/RECERRMAX/auc	OoD/RECERRMEAN/auc

abmil	0.899 ± 0.028	0.867 ± 0.046	-	-	-	-
clam	0.726 ± 0.071	0.722 ± 0.070	-	-	-	-
daeabmil	0.891 ± 0.027	0.916 ± 0.027	-	-	0.848 ± 0.006	0.970 ± 0.008
dftdmil	0.803 ± 0.049	0.790 ± 0.053	-	-	-	-
dsmil	0.878 ± 0.010	0.864 ± 0.003	-	-	-	-
transmil	0.877 ± 0.040	0.836 ± 0.072	-	-	-	-
vaeabmil	0.882 ± 0.035	0.877 ± 0.022	0.828 ± 0.027	0.959 ± 0.003	-	-
(c) ood detection results for the different ood scores in the (panda, cam16) experiment.						
Model	OoD/Entropy/auc	OoD/MLS/auc	OoD/LOGPXMAX/auc	OoD/LOGPXMEAN/auc	OoD/RECERRMAX/auc	OoD/RECERRMEAN/auc

abmil	0.959 ± 0.007	0.956 ± 0.005	-	.	-	-
clam	0.697 ± 0.052	0.654 ± 0.045	-	-	-	-
daeabmil	0.333 ±0.156	0.334 ±0.156	-	-	0.999 ± 0.000	1.000 ±0.000
dftdmil	0.944 ± 0.030	0.949 ± 0.025	-	-	-	-
dsmil	0.793 ± 0.031	0.833 ± 0.032	-	-	-	-
transmil	0.911 ±0.022	0.888 ± 0.033	-	-	-	-
vaeabmil	0.965 ± 0.016	0.982 ± 0.013	1.000 ±0.000	1.000 ± 0.000	-	-
(d) ood detection results for the different ood scores in the (panda, artif) experiment.						
Model	OoD/Entropy/auc	OoD/MLS/auc	OoD/LOGPXMAX/auc	OoD/LOGPXMEAN/auc	OoD/RECERRMAX/auc	OoD/RECERRMEAN/auc

abmil	0.755 ± 0.025	0.771 ± 0.027	-	-	-	-
clam	0.679 ± 0.028	0.646 ± 0.029	-	-	-	-
daeabmil	0.327 ± 0.076	0.344 ± 0.088	-	-	0.998 ± 0.001	0.999 ± 0.000
dftdmil	0.689 ± 0.029	0.704 ± 0.036	-	-	-	-
dsmil	0.541 ± 0.039	0.566 ± 0.037	-	-	-	-
transmil	0.698 ± 0.041	0.688 ± 0.049	-	-	-	-
vaeabmil	0.738 ± 0.026	0.771 ± 0.036	1.000 ±0.000	0.999 ± 0.000	-	-

## Footnotes

1 Link to Camelyon16’s challenge.

2 Link to Panda’s challenge.

3 CLAM’s code in GitHub.

4 Remark that the rest of the models can not predict instance-level ood scores.

## APPENDIX A

### STATISTICAL SIGNIFICANCE TEST

To assess whether differences in model performance in the ood detection task are statistically significant, we employ the paired t-test, a parametric test designed to compare two related samples [56]. In our case, since we executed n=3 train/test partitions per model, we compare the ood detection auc of each model in each partition with the results of rest of the models in the same partition. The paired t-test evaluates whether the mean difference between these paired scores is significantly different from zero under the assumption that the differences are normally distributed. To achieve this, we compute the statistic $\text{t\_stat}\;\text{=}\;\bar{d}\;\cdot \;\sqrt{n}\text{/}s_{d}$, where $\;\bar{d}$ is the mean difference of the results in each of the n splits and $s_{d}$ is the standard deviation of the differences. This test is ideal here because it accounts for the dependencies between the two sets of scores by considering that they were computed on the same data partitions.

Tables 1, 2, 3, and 4 show the results of comparing both vaeabmil and daeabmil with current sota models across the different performed experiments using the uni feature extractor. Using a level of significance of 0.05, the results show that:

- The differences between the results of vaeabmil and daeabmil are not significant in any case. This highlights the fact that, when using uni features, both models perform equally at detecting ood samples.
- In the (panda, artif) experiment, the differences between our proposals and the sota models are always significant, as shown in Table 4. The reason for this is that those methods do not model the data distribution, making post-hoc ood scores worse in this scenario. Furthermore, the artifacts are, in proportion, much smaller than the main tissue, as shown in Figure 15.
- In the (cam16, panda), (cam16, bcell) and (panda, cam16) experiments, some of the sota models obtain non-significant differences according to the paired t-test. However, we observe that the standard deviation of the ood/auroc in our models is much smaller than in the rest of the models. Hence, we state that if the high variance was maintained when increasing the number of executions, the results would change to obtain significant differences between our proposals and the sota mil models.

## APPENDIX B

### RESULTS WITH ALL THE ood SCORES

To provide a comprehensive analysis of the performance of the different ood scores across the used models, we present the complete results in Table 5. The results show that, for abmil, transmil, and clam, the Entropy score obtained the highest ood detection results in all cases. In dtfdmil, the mls achieves the best results except in the (cam16, bcell) scenario. In dsmil, the Entropy is a better ood detector when the ind dataset is cam16, and the mls performs better when the ind dataset is panda.

Regarding the proposed models, we observe that using the mean aggregator yields better results than the max aggregator in all cases except for the (panda, artif) experiment. The difference between the aggregator is, however, negligible in that case. In other cases, such as when considering cam16 as the ind dataset, the max aggregator struggles to detect ood samples while applying the mean aggregator provides much better results.

In summary, when using the mean aggregator for logpx in vaeabmil and for recerr in daeabmil, the proposed models obtain the best performance in the ood detection task.
